# Supplementary material for: Characterization Standard for In-situ Cryo-electron Tomography
Source: bioRxiv. 2026 May 22:2026.05.20.726049. Preprint. [Version 1] doi: 10.64898/2026.05.20.726049 (PMC13228412; doi:10.64898/2026.05.20.726049)
Supplement: Supplement 1 [file NIHPP2026.05.20.726049v1-supplement-1.pdf]

bioRxiv preprint doi: https://doi.org/10.64898/2026.05.20.726049; this version posted May 22, 2026. The copyright holder for this preprint (which was not certified by peer review) is the author/funder, who has granted bioRxiv a license to display the preprint in perpetuity. It is made available under a CC-BY 4.0 International license.

.

Supplementary Tables

Sample Type	Plunge-frozen		FIB-milled	
Tilt Series	Position_142.mrc	Position_58.mrc	Position_9_11.mrc	Position_9_2.mrc
Thickness (nm)	2190	2160	2190	2160
Tilt Axis	-96.06	-96.50	-96.52	-96.03
Global Shift (pix)	76.50	246.94	140.11	95.69
Bad Patch All	0.00	0.00	0.00	0.00
Bad Patch Low	0.00	0.00	0.00	0.00
Defocus (Å)	27704	27638	25093	24052
CTF-Res (Å)	7.92	7.92	8.00	7.68
CTF-Score	0.21	0.22	0.19	0.19
Alpha0	-2.20	4.20	22.80	22.80
Beta0	-11.20	3.00	-3.00	-12.30
Particles	1218	1082	2206	2003

Supplementary Table 1 | AreTomo3 metrics for the two-tomogram tests for the plunge-frozen and high-pressure frozen datasets. Tomograms were selected to have broadly similar metrics such as estimated thickness, CTF fit resolution and score, and number of bad patches from AreTomo3 local alignments. Alpha0 and Beta0 represent the tilt offset about the tilt axis and orthogonal axis, respectively.

bioRxiv preprint doi: https://doi.org/10.64898/2026.05.20.726049; this version posted May 22, 2026. The copyright holder for this preprint (which was not certified by peer review) is the author/funder, who has granted bioRxiv a license to display the preprint in perpetuity. It is made available under a CC-BY 4.0 International license.

Milling Step	Beam Current (Xe)	Pattern Type	Time/Depth
Trench Milling	60 nA	Rectangle[1]	~30-90 s per trench
Undercut Milling	4.0 nA	Rectangle	~15 s per milling angle (40,30,20)
Notch Milling	1.0 nA	Rectangle[2]	120 s
Rough Milling	1.0 nA	Rectangle	200% Depth Correction
Medium Milling	1.0 nA	Rectangle	200% Depth Correction
Fine Milling	0.1 nA	Rectangle	150% Depth Correction
Polishing 1	30 pA	Cleaning Cross Section	100% Depth Correction
Polishing 2	10 pA	Cleaning Cross Section	70% Depth Correction

[1] Special Pattern Dimensions - Two rectangles placed 30.00 μm apart: x = 20.00 μm, y = 35.00 μm.

[2] Special Pattern Dimensions - Five rectangles: vertical rectangles - x = 0.25 μm, y = 10.00 μm, horizontal rectangles - x = 0.25 μm, y = 3.50 μm

Supplementary Table 2 | Table of beam currents, patterns, and run times used for each milling step. Waffle milling steps and corresponding beam currents, pattern type, and milling time & depths are listed. Patterns for trench, undercut, and notch milling were manually drawn with the special pattern dimensions listed. Undercut milling was completed by drawing and milling rectangles at three decreasing angle intervals (40, 30, and 20 degrees) to remove the ice until ~3.5um remained. Patterns for the remaining milling steps were presets in the AutoTEM software. Additionally, 'polishing 2' was run two supplemental times after the automated steps were completed to ensure the lamella had a smooth and consistently thin appearance.

Parameter	PF 25oct20a	PF 25oct20a (run2)	HPF 26feb20d
Align	1	1	1
AlignZ	0	0	0
AmpContrast	0.07	0.07	0.07
AtPatch	[4, 4]	[4, 4]	[3, 3]
CorrCTF	[1, 15]	[1, 15]	[1, 15]
Cs	2.7	2.7	2.7
DarkTol	0.7	0.7	0.7
EerSampling	2	2	2
FlipGain	1	1	1
FlipVol	1	1	1
FmInt	10	15	15
Group	[2, 4]	[2, 4]	[1, 3]
McBin	2.0	2.0	2.0
McIter	15	15	15

bioRxiv preprint doi: https://doi.org/10.64898/2026.05.20.726049; this version posted May 22, 2026. The copyright holder for this preprint (which was not certified by peer review) is the author/funder, who has granted bioRxiv a license to display the preprint in perpetuity. It is made available under a CC-BY 4.0 International license.

**McPatch**	**[4, 4]**	**[4, 4]**	**[4, 4]**
**McTol**	**0.1**	**0.1**	**0.1**
**OutImod**	**1**	**1**	**1**
**PixSize**	**1.5**	**1.5**	**1.5**
**ReconRange**	**[-90.0, 90.0]**	**[-90.0, 90.0]**	**[-90.0, 90.0]**
**SplitSum**	**1**	**1**	**1**
**TiltAxis**	**[0.0, 1.0]**	**[0.0, 1.0]**	**[0.0, 1.0]**
**TiltCor**	**[0.0, 0.0]**	**[1.0, 0.0]**	**[1.0, 0.0]**
**VolZ**	**1600**	**1600**	**1600**
**Version**	**v2.7.0**	**v2.3.0**	**v2.3.0**

Supplementary Table 3 | List of AreTomo3 parameters for motion correction and tilt series alignments for the different datasets. PF 25oct20a was used as input for particle picking and subtomogram averaging. PF 25oct20a (run2) was only used for thickness and CTF values distribution in comparison with HPF 26feb20d. HPF 26feb20d was used for particle picking and STA.
